# Experimental study of the bond behavior of 400MPa grade hot-rolled ribbed steel bars in steel fibre reinforced concrete

**DOI:** 10.1038/s41598-024-54703-8

**Published:** 2024-02-19

**Authors:** Minglei Zhao, Jie Li, Yi Min Xie, Jianhu Shen, Changyong Li

**Affiliations:** 1 School of Engineering, RMIT University, Melbourne, 3001 Australia; 2 International Joint Research Lab for Eco-Building Materials and Engineering of Henan, North China University of Water Resources and Electric Power, Zhengzhou, 450045 China

**Keywords:** Engineering, Materials science

## Abstract

Present studies show that steel fibres can improve the bond of steel bar in steel fibre reinforced concrete (SFRC) with a correlation to the fibre factor and the fibre distribution uniformity. As a foundation of high-flowability SFRC working together with 400 MPa grade hot-rolled ribbed (HRB400) steel bar in reinforced structures, the bond between them was evaluated through a series of pull-out testing on 48 specimens with a central arranged steel bar. The bond behaviours of steel bar were estimated with a constant bond length of 5*d* (*d* is the diameter of steel bar) embedded in high-flowability SFRC, the main research parameters included the ingot mill steel fibres with a fibre volume fraction varied from 0.8 to 2.0%, the strength grade C40 and C50 of SFRC or referenced conventional concrete, and the diameter of steel bars varied from 14 to 20 mm. Results showed that the high-flowability SFRC compacted with a slight vibration is beneficial to improve the bond failure pattern since steel fibres effectively eliminate the crack appeared on the SFRC blocks during the pulling out of steel bar, leading to all specimens failed with the steel bar pull out of SFRC blocks. The bond strength was dominant by the SFRC strength, and obviously strengthened with the increase of fibre volume fraction, while the peak-slip was slightly influenced by the diameter of steel bar. By conducting analyses of test data, equations for calculating the bond strength and the peak-slip are proposed accounting for the effect of steel fibres. Then the predicting method for the anchorage length is suggested linking with different design codes for concrete structures. Compared with test results of this study, a little shorter anchorage length of steel bar in SFRC is obtained from the specification of Chinese code JGJ/T46, which should be noticed to ensure a rational anchorage of ribbed steel bar in SFRC with ingot mill steel fibres.

## Introduction

For the concrete structures reinforced with steel^1,2^ or FRP^3^, the bond of steel bar, formed steel or FRP with concrete is a basic property that ensures their integrated resistance to internal or external actions. Based on the mechanism of the bond behaviour between ribbed steel bar and concrete, the chemical adhesion of steel bar mainly contributes to the bond before slipping, the friction on rough surface of steel bar acts in the initial stage of slipping, and the mechanical interaction force between compressive side of ribs and concrete is dominant when the slipping occurs^4,5^. This relies on many factors including the interfacial quality between steel bar and concrete, the surface roughness and morphology of steel bar, the physical dimension of ribs, the constituents and strength of concrete, as well as the bond length, the position of steel bar in concrete, and the thickness of the concrete cover^3,5–7^. Therefore, the bond of steel bar with concrete is a complex issue. It is critical to identify the bond behaviour in different combinations of these factors for the real arrangement of steel bars in reinforced concrete structural members^1,3^. Commonly, a classical method of pull-out test is used for measuring the bond behaviour of a steel bar centrally embedded in concrete blocks under a central tensioning force^3,8,9^. To get a complete bond stress-slip curve of the steel bar pulled out of the concrete block, enough thickness of concrete cover or additional stirrups are required to prevent the splitting failure created by the radial component force on the interface between ribs and concrete^10–12^.

As is known, steel fibre reinforced concrete (SFRC) can markedly strengthen the concrete structural elements in flexural or shear^13,14^. This encourages the innovative investigations using SFRC as a hybrid portion to effectively strengthen the flexural performance or the shear resistance of reinforced concrete beams^15–17^. As far as the central pull-out test for the bond of steel bar in concrete, the splitting failure of concrete blocks can be overcome when the conventional concrete is changed to be SFRC^4^. Because the effective reinforcement of steel fibres on the tensile strength of concrete prevents the microcracking of concrete on the bonding interface and provides a passive confinement to the transversal expansion of concrete block, the steel bar can be centrally pulled out of non-splitting SFRC to measure the post-peak bond behaviour with a ductile descending portion of bond stress-slip curve^18,19^. Yazici and Arel^20^ reported that for the deformed steel bar with a diameter of 14 mm, embedded in grade C20–C60 SFRC, the bond pull-out loads had an increasement of 16% with the steel fibre content increased from 10 to 80 kg/m^3^. Garcia-Taenga et al.^21^ conducted an experimental investigation on the bond of deformed steel bar with diameters of 8–20 mm in SFRC with compressive strength of 30–50 MPa, considering the variations of the concrete cover, and the fibre content, length and aspect ratio. Results indicated that a higher bond strength was yielded with a higher concrete compressive strength and a larger steel bar diameter. Limited effect of fibre content was presented on the bond strength, while more effective improvement could reach with shorter fibers than longer ones with the same fibre content. Chu and Kwan^22^ proposed a bond model for steel bar in SFRC by modifying the existing one for concrete in *fib* MC 2010^23^, in which a synergistic effect of concrete strength and steel fibres is found on the bond performance. The study of Li et al.^24^ indicated that the combined addition of steel fibres and expansive agent would substantially improve the bond strength and stiffness of steel bar in SFRC. More importantly, such combined addition would offer the synergistic effects over that obtained by the separately addition. Hou et al.^25^ conducted an extensive study on the bond strength of steel bar in SFRC considered multi factors, including bond test methods, the cover to steel bar diameter ratio, the bond length to steel bar diameter ratio, the fibre content and aspect ratio, and the steel bar diameter. Results indicated that, the cover to steel bar diameter ratio and the bond length to steel bar diameter ratio have great effect on the bond strength, while the effect of steel fibre is correlated with the fibre factor and fibre distribution uniformity. The study of de Alencar Monteiro et al.^26^ exhibited that the steel fibres provide a post-crack resistance to the composite to raise the confinement around the interface, and significantly increased the bond strength, stiffness and toughness, while the fatigue life of cyclic pull-out tests could be successfully increased with the addition of steel fibres. In summary, ribbed steel bars embedded in SFRC behave a better bond performance with an enhanced bond strength at the decreased slip, and improved the ductility of the bond failure. This attributes to the reinforcement of steel fibres, not only on the tensile strength, but also on the compressive strength of SFRC^19,27^. Fibers improve concrete bond capacity by confining the bars, and their role is similar to that of stirrups. Also, by widening the crack widths’ range within, this confinement remains active. In terms of bond capacity, this improvement can be regarded as a consequence of the betterment of matrix properties due to the fibers. As a result, the using of SFRC contributes to reduce the anchorage length of steel bar in SFRC structures. This is specified in Chinese code JGJ/T465^28^, in which the tensile strength of conventional concrete is replaced by that of SFRC for the calculation of anchorage length.

With the development of high-flowability SFRC, the fibres distribution and orientation in concrete matrix and the mechanical properties have been thoroughly investigated considering the influences of fresh flowability, the properties of raw materials and their mix proportion, and the compaction method^29,30^. The high-flowability SFRC are featured with the production with a higher content of binders with mineral admixtures, a small particle size of coarse aggregate, and the tendency of horizontal oriented distribution of the steel fibres in specimens compacted using a slight vibration. There are potential influences on the bond performance of steel bars. In this aspect, Zhang et al.^31^ pointed that the bond behavior of deformed steel bar in SFRC is significantly improved by oriented fibers, due to the interlocking between the steel bar ribs and steel fibers near the steel bars for the case without splitting failure. This positive effect on the bond behavior increased with more steel fibers distributed near the steel bars, and stiffer of these fibers. Therefore, it is necessary to correctly evaluate the bond of high-flowability SFRC with steel bars to ensure their working together in reinforced concrete structures.

In this paper, an experimental study was carried out for the bond performance of the 400 MPa grade hot-rolled ribbed (HRB400) steel bar embedded in high-flowability SFRC. Forty-eight specimens, divided into 24 groups, with a centrally arranged steel bar were prepared and tested using a central pull-out test method. The volume fraction of ingot mill steel fibre with a varying from 0.8 to 2.0% was considered to evaluate the contribution of high-flowability SFRC to the bond with HRB400 steel bars. Meanwhile, the influences of the strength of SFRC and the conventional concrete, and the diameter of steel bar, on the bond of HRB400 steel bars embedded in high-flowability SFRC were also experimentally studied. The loads and displacements were measured to determine the complete bond stress-slip curve of each specimen. And then the test results are analyzed on the failure pattern of specimens, and the effects of test factors on the bond behaviour of HRB400 steel bar embedded in high-flowability SFRC. Equations for predicting bond strength and slip accounting for the effect of steel fibres are proposed. Finally, linked with the specifications in codes^23,32,33^, a prediction equation for the anchorage length of steel bar embedded in high-flowability SFRC is suggested.

## Experiment work

### Preparation of high-flowability SFRC

**Figure 1:**
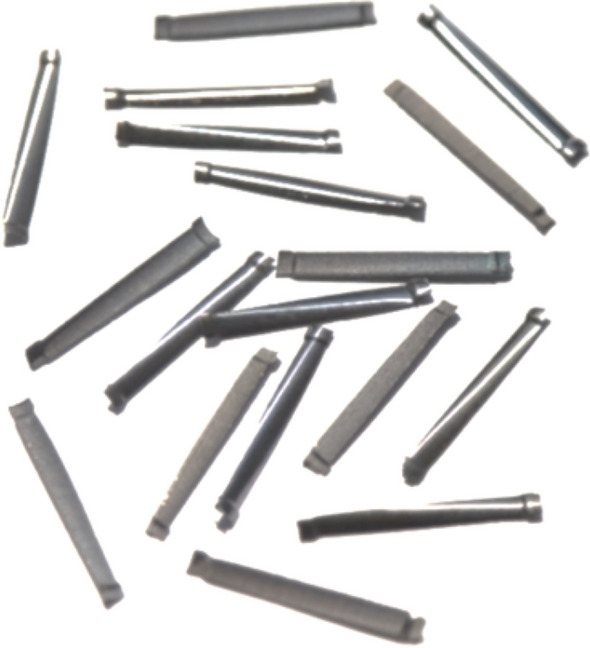
Ingot mill steel fibres used in this study.

Raw materials used in this study included Grade 42.5 common Portland cement, Class-II fly ash, natural river sand, continuous grading crushed limestones, ingot mill steel fibre, and superplasticizer (water reducing agent). Physical and mechanical property of each material were tested according to related Chinese codes^34^. The cement was 3040 kg/m^3^ in density, with an initial setting time of 170 min and a compressive strength of 44.2 MPa at a curing age of 28 days, which met the requirement of common Portland cement specified in Chinese code GB 175^35^. The fly ash was , which met the requirement of fly ash for concrete specified in Chinese code GB/T 1596^36^. The river sand was 2610 kg/m^3^ in apparent density and 1653 kg/m^3^ in closed volume density; the crushed limestone was continuous grading with a maximum particle size of 10 mm, 2800 kg/m^3^ in apparent density, 1615 kg/m^3^ in closed volume density, and a crushed index of 10.8%. They met the requirement of sand and crushed stone for concrete specified in Chinese code JGJ52^37^. The ingot mill steel fibre was produced with an average length *l*_f_ = 32 mm, an equivalent diameter *d*_f_ = 0.8 mm, and an aspect ratio *l*_f_/*d*_f_ = 40. As shown in Fig. 1, it met the requirement of Chinese code JG 472^38^.

The SFRC mix design was developed based on volumetric method similar to the mix design of conventional concrete^38,39^. A direct mix design method was used, in which the coarse aggregate was replaced by an equal-volume of steel fibre, and the mass of steel fibre was considered in the calculation of sand ratio^40^. The slump of fresh mix was designed over 150 mm. A water reducing agent was used to increase the slump of the mixture without changing *w/b* and the amount of water used. The volume fraction of steel fiber *v*_f_ = 0, 0.8%, 1.2%, 1.6% or 2.0% was used for producing the high-flowability SFRC.

**Figure 2:**
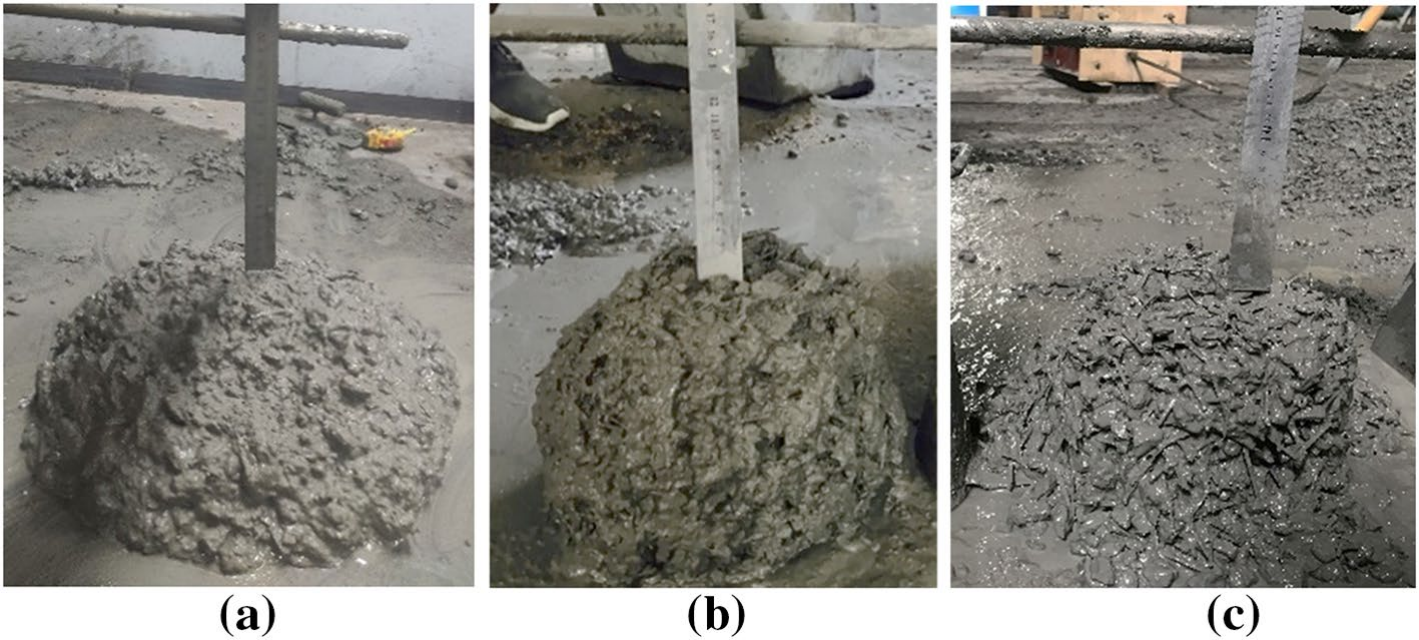
Slump flow test. (**a**)* v*_f_=0.8% (**b**) *v*_f_=1.6% (**c**) *v*_f_=2.0%.

A horizontal shaft mixer was used to mix the fresh SFRC. Crushed limestones, sand followed by adding cement and fly ash was mixed for 1 min, then water was added for continue mixing of 50 s. Finally, the remaining water and the water reducing agent were added for mixing of 3 min. Special attention was paid to the state of the fresh mix during the mixing process, steel fibres must be evenly spread into the stirred concrete, and the mixing time was strictly controlled to limit the early hydration of cement. The working performance of fresh mixes was timely measured after mixing, using the slump cone method specified in Chinese code GB50080^41^. As shown in Fig. 2, the slump of fresh SFRC appeared a decrease from 200 to 160 mm when the fibre volume fraction increased from 0.8% to 2.0%. This corresponds to the high-flowing concrete with a slump over 160 mm as specified in Chinese code JGJ55^39^. Therefore, the fresh SFRC in this study has a characteristic of high-flowability that distinguishes from that with low slump. Actually, a flowing feature of the fresh SFRC was apparent when compacted with a slight vibration. Therefore, the fresh mixes could meet the designed compaction quality of specimens with uniformly distributed fibres.

**Figure 3:**
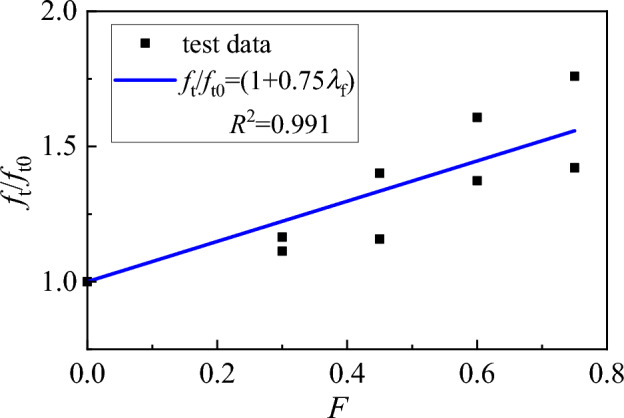
Relationship between splitting tensile strength and fibre factor.

In order to obtain the actual mechanical properties of SFRC for the bonding specimens, the basic mechanical properties of the accompanied specimens were tested first in accordance with the Chinese code GB/T50081^9^. For each test category, three ϕ150 mm × 300 mm cylinders were casted for axial-compressive strength test, three ϕ150 mm × 300 mm cylinders for elasticity modulus test, three cubes with a dimension of 150 mm for cubic compressive strength test, and three cubes with a dimension of 150 mm for splitting-tensile strength test. Test results are presented in Table 1. It can be seen that all the mechanical properties of SFRC including the compressive strength, the splitting tensile strength, and the modulus of elasticity, increased with the fibre content. This is consistent to previous studies of high-flowability SFRC^29,30^. As shown in Fig. 3, a linear relationship is built for the splitting tensile strength with a fibre factor by a fitness of test data, and can be predicted using the following equations,

1 $$f_{{\text{t}}} { = }\left( {1 + 0.74F} \right)f_{{{\text{t0}}}}$$

2 $$F{ = }\left( {\frac{{l_{{\text{f}}} }}{{d_{{\text{f}}} }}} \right)v_{{_{{\text{f}}} }} \eta_{{\text{f}}}$$

**Table 1:** Test results of mechanical properties of concrete with different fibre content.

Strength grade	*v*_f_ (%)	Cubic compressive strength *f*_cu_ (MPa)	Axial compressive strength *f*_c_ (MPa)	Splitting tensile strength *f*_t_ (MPa)	Modulus of elasticity *E*_c_ (× 10^4^ MPa)
C40	0	40.4	33.3	2.37	3.25
	0.8	46.2	37.4	2.76	3.93
	1.2	45.9	41.0	3.32	4.42
	1.6	50.3	41.2	3.81	4.68
	2.0	53.7	45.8	4.17	4.58
C50	0	46. 7	38.2	3.11	3.78
	0.8	48. 9	39.6	3.46	4.51
	1.2	61.2	48.4	3.60	4.92
	1.6	57.3	48.9	4.27	4.89
	2.0	60.4	49.1	4.42	5.24

In which the fibre bonding factor *η*_f_ = 1.0 for ingot mill steel fibre^34^.

### Steel bars

The HRB400 hot-rolled ribbed steel bars of 400 MPa grade was used in this test, the measured mechanical properties and physical dimensions are summarized in Table 2.

**Table 2:** Mechanical properties and physical dimensions of steel bars.

Diameter *d *(mm)	Yield Strength *f*_y_ (MPa)	Ultimate tensile strength *f*_st_ (MPa)	Elongation after break *δ *(%)	Inner diameter (mm)	Ribs (mm)		
					Height	Longitudinal	Spacing
14	425.2	572.6	30.8	13.36	1.17	1.14	8.54
16	436.9	569.1	25.8	15.22	1.24	1.39	10.48
20	440.7	593.9	27.4	19.04	1.33	1.56	10.61

### Specimens for pull-out tests

**Figure 4:**
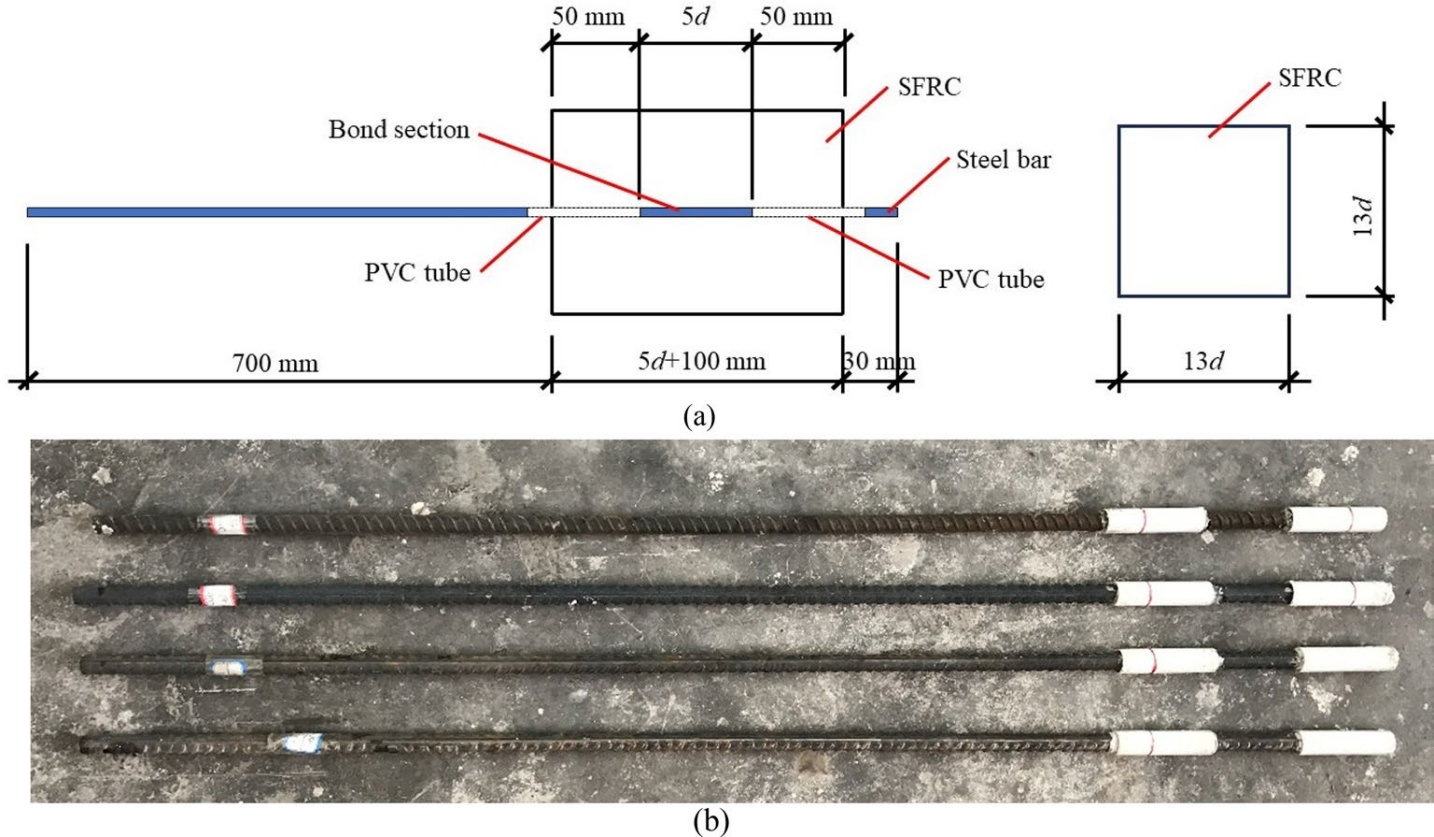
Specimen for bond strength test. (**a**) schematic of specimens (**b**) Treated steel bars.

To eliminate the boundary influence at the free and tension ends of SFRC block on the bond behavior of ribbed steel bar, the bond length was set at the central of specimens in this study. As shown in Fig. 4, the bond length *l*_b_ = 5*d*, the free length was 50 mm at the free and tension ends sheathed by a PVC tube, and the length of the SFRC block for bonding test was 5*d* + 100 mm. A square section with a dimension over 13*d* (*d* = diameter of steel bar) was designed for the SFRC block, which was set to prevent a splitting failure of block with a sufficient thickness of concrete cover over 6*d*^4,9^. Therefore, the specimens, with the steel bar of a diameter of 14 mm, 16 mm, and 20 mm, had a length of 170 mm, 180 mm, and 200 mm, with a sectional dimension of 200 mm, 300 mm, and 300 mm, respectively. Correspondingly, the bond length was 70 mm, 80 mm, and 100 mm, respectively. Two same specimens were made for each group. Forty-eight specimens for 24 groups in total were prepared for testing. Details are presented in Table 3.

**Table 3:** Details of test specimens.

Trial no.	SFRC			Steel bar			Number of specimens
	Strength grade	*v*_f_ (%)	Block sectional size × length (mm)	Strength grade	*l*_b_ (mm)	Total length (mm)	
C4H4/14-0	CF40	0	200 × 170	400	70	900	2
C4H4/14-0.8		0.8					2
C4H4/14-1.2		1.2					2
C4H4/14-1.6		1.6					2
C4H4/14-2.0		2.0					2
C4H4/20-0	CF40	0	300 × 200	400	100	930	2
C4H4/20-0.8		0.8					2
C4H4/20-1.6		1.6					2
C4H4/20-2.0		2.0					2
C5H4/14-0	CF50	0	200 × 170	400	70	900	2
C5H4/14-0.8		0.8					2
C5H4/14-1.2		1.2					2
C5H4/14-1.6		1.6					2
C5H4/14-2.0		2.0					2
C5H4/16-0	CF50	0	300 × 180	400	80	910	2
C5H4/16-0.8		0.8					2
C5H4/16-1.2		1.2					2
C5H4/16-1.6		1.6					2
C5H4/16-2.0		2.0					2
C5H4/20-0	CF50	0	300 × 200	400	100	930	2
C5H4/20-0.8		0.8					2
C5H4/20-1.2		1.2					2
C5H4/20-1.6		1.6					2
C5H4/20-2.0		2.0					2

Due to the specimen dimensions changed with the diameter of steel bars, the assembly of steel and wood formworks were used for the moulds of specimens. The wood plate was customized according to the size of the height and width of the block with a hole drilled in the center to penetrate the PVC sleeve for the steel bar. As the length of the bonding block was different with different diameters of steel bar, the distance between the wood plates was changed to meet the test requirements. A pair of bolts were used on the top to fix the distance of plates, while the corners at the bottom of the plates were all fixed. The blocks were compacted on a vibration platform with a slight vibration, smoothed with a spatula for its surface, and covered with the plastic film. The demould was carried out after 1 to 2 days. And then, the wet quilt was covered on the specimens to ensure a wet curing condition for 28 days.

### Pull-out test method

**Figure 5:**
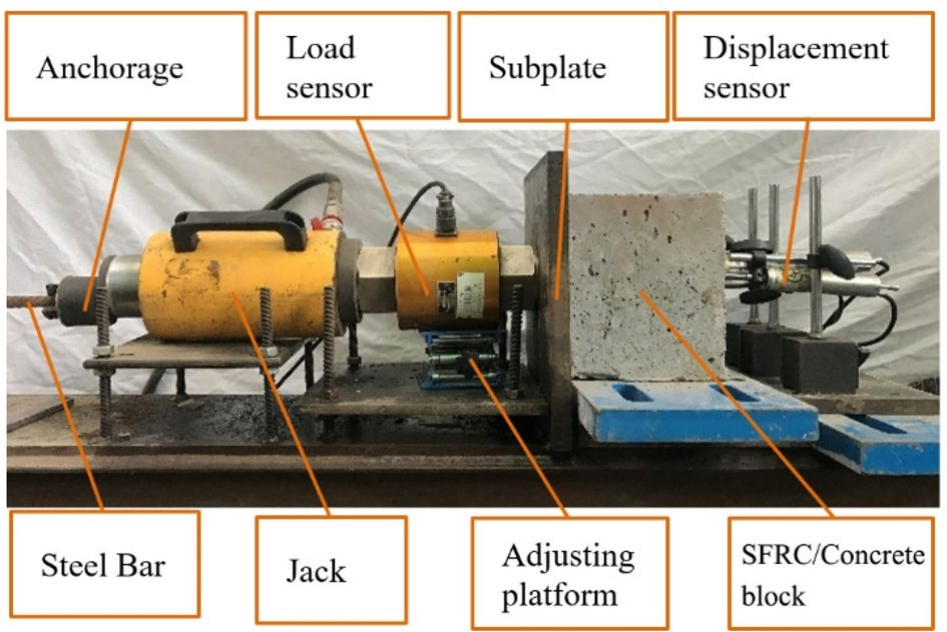
Composition of the pull-out testing device.

In this study, the pull-out test was carried out on a horizontal tensioning device^9,42^. The composition of test device is exhibited in Fig. 5, two lift platforms on pedestal were used to support the load sensor and jack. The lift platforms could adjust the centers of load sensor and jack to be on the same axis, as per the required height of loading point fitted to different specimen sizes. Meanwhile, a steel connector was used with one convex side inserted in the jack, and the other groove side to fix the load sensor. This ensures the close link of the jack with the load sensor without tilt. A steel subplate was fitted on the contact surface of bonding block to ensure no local deformation in the process of loading. The inner diameter of the holes of jack, load sensor and subplate ensured that the steel bar could be passed smoothly. In general, the test device is able to adjust the platform with fine-tuning to improve the test efficiency, and reduce the possible errors of the test results generated in the test operation process.

**Figure 6:**
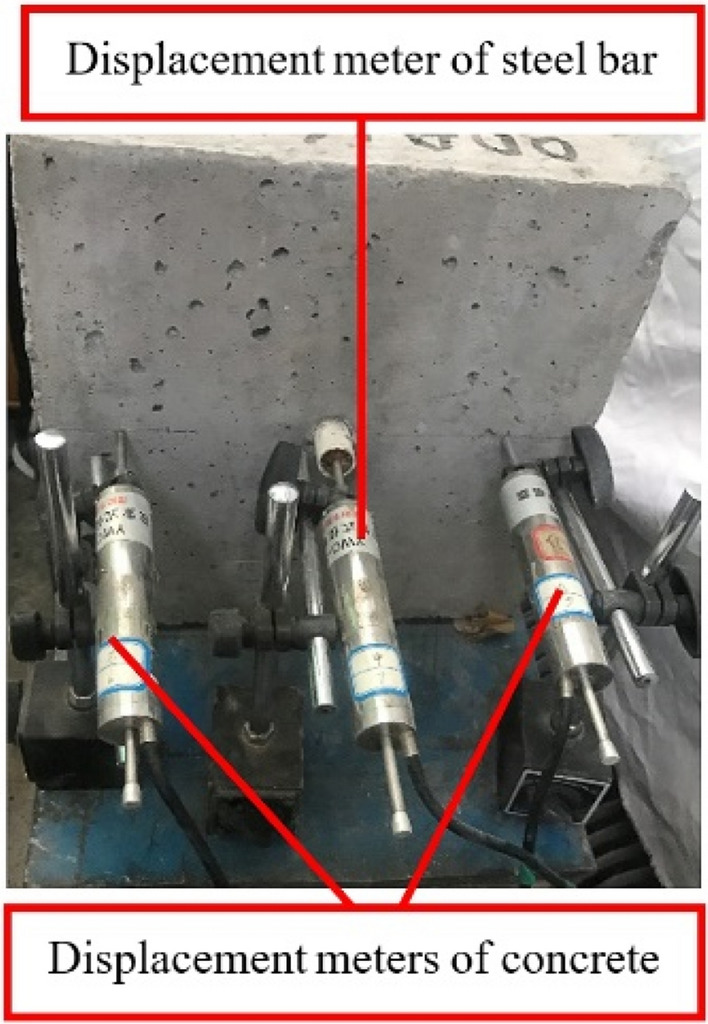
Displacement meters arrangement at free end of steel bar and on the surface of block.

The data acquisition system used in this test category was Donghua DH-3821 static strain test system. Four channels were needed to respectively connect the load sensor, the steel bar displacement meter and the concrete displacement meters on both sides of the steel bar, as shown in Fig. 6.

The following steps were used to carry out the tests:

1. Put the bonding specimen on the loading platform. Pass the load sensor, connector, jack and anchorage through the steel bar in turn. Adjust the lifting platform and fine-tuning platform to ensure the sensor and hollow self-reset hydraulic jack in the same horizontal axis. And then, use the jack to pre-tensioning the specimen with a magnitude of 3–5 kN to make a close contact among the components of loading device.
2. Install the displacement meters at the predetermined position. Balance the data collector to ensure the success of assembling of each link.
3. Apply the tensile force on steel bar, collect and save testing data. Observe and record carefully about any abnormality during the test.
4. Unscrew the oil pump valve first after the test, then remove the displacement meters and the loading device. In the end, collect the bonding specimen to observe the surface and take photos for future reference.

The average bond-stress was calculated as follows:

3 $$\tau { = }{P \mathord{\left/ {\vphantom {P {\pi dl_{{\text{b}}} }}} \right. \kern-0pt} {\pi dl_{{\text{b}}} }}$$

The peak bond-stress was the bond strength, while the slip was the peak-slip at the bond-slip curve. Since the bond length of this test was relatively short, the bond-slip was regarded as the slip amount of the free end of the steel bar^9^. Because the rib spacing is different for the steel bar with different diameters, a reliable bond-slip was taken as one time of the diameter of the steel bar in this test category.

## Analysis of test results

### Failure pattern of bonding specimens

At the early stage of tensioning, the first response of bonding surface between steel bar and SFRC or conventional concrete was to overcome the cementation cohesiveness, while the sliding hardly changed. With the increase of tensile force, a small displacement increased at the free end when the steel bar began to slide, and the slip of steel bar could be measured by the displacement meters. After that, the slip measured by the displacement meters gradually increased, until the steel bar was slowly pulled out. In this test category, the ratio of the thickness of concrete cover to the diameter of steel bar was over 6, which eliminated the possibility of splitting damage^4,21^. After the pull-out test, no crack appeared on SFRC blocks, while that occurred on some of conventional concrete ones. Except for the steel bars of two groups of specimens entered the stress strengthening stage, others did not yield.

**Figure 7:**
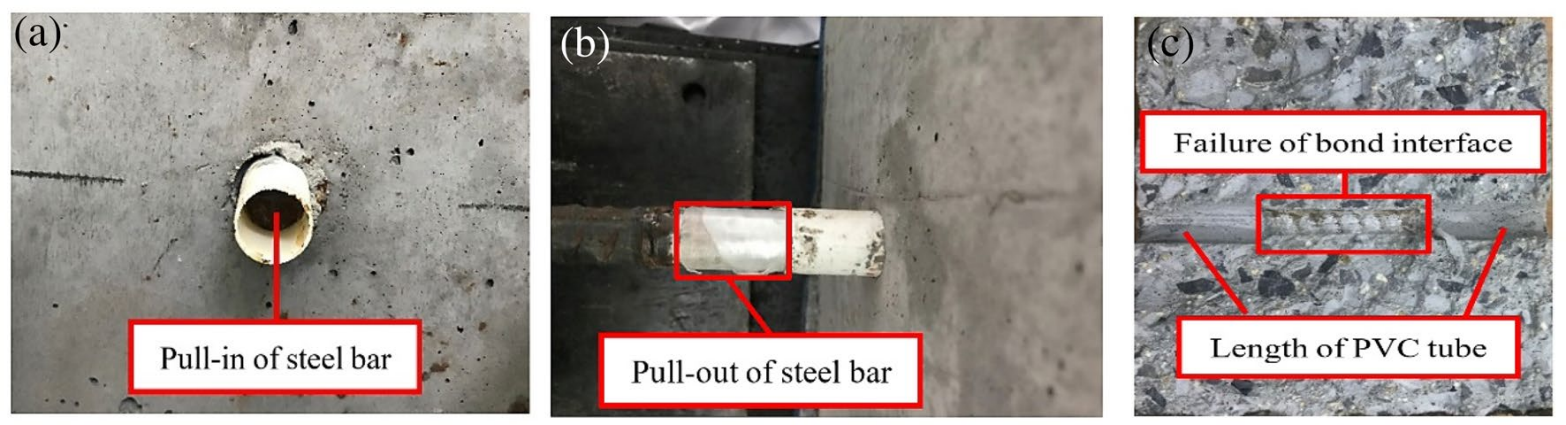
Typical failure patterns of tested specimens. (**a**) pull-in at free end (**b**) pull-out at tension end (**c**) failure surface on bond length.

As all specimens failed in pull-out pattern without splitting of blocks, a group of pictures is typically exhibited in Fig. 7. It is easily observed the pull-in of steel bar at the free end from Fig. 7a), and the pull-out of steel bar at the tensile end from Fig. 7b). With the continuously increase of tensile force, the steel bar was gradually pulled out accompanied with the sound of steel fibre tearing occurred inside the specimen. From the post-split section of tested specimen along the steel bar as shown in Fig. 7c), it is found that the SFRC was obviously squeezed between the ribs of steel bar, and the crushed SFRC was remained along the bond interface.

### Effect of steel bar diameter

**Figure 8:**
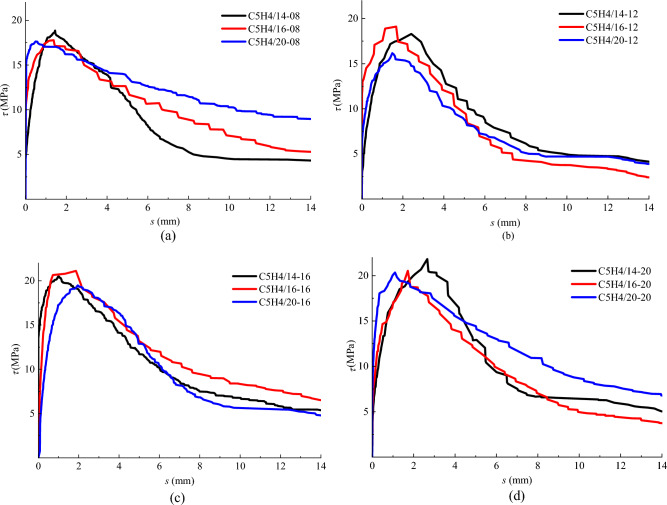
Bond stress-slip curves of specimens with different diameter of steel bar. (**a**) *v*_f_=0.8% (**b**) *v*_f_=1.2% (**c**) *v*_f_=1.6% (**d**) *v*_f_=2.0%.

As is known, the bond strength comes from the chemical adhesiveness before the slip takes place, and from the surface friction between steel bar and concrete with the occurring of the slip. This makes the same initial ascending bond stress-slip curve with a difference came from the different interfacial roughness that controls the surface friction between steel bar and concrete. Because the bond stress is calculated with Eq. (1), the effect of steel bar diameter can be fully accounted for in this calculation, resulting in a neglection of the influence of steel bar diameter on the bond-stress in theory. This is rational for the plane steel bar in concrete, while the issue becomes complex for the ribbed steel bar in concrete, since different bonding mechanism appeared after the sliding of steel bar^4,5^. The inconsistent results to the theoretical law discussed above are reflected on the bond-slip curves of specimens changed with the diameter of steel bar, as shown in Fig. 8. In which the specimens are divided into a group with the same fibre volume fraction and concrete strength.

With the increase of sliding, the interlocking effect of the ribs of steel bar plays a dominant role to impede the slip of concrete in front of the compression side of the ribs^6,10^. This blocking effect will be relied on the physical dimension of steel bar especially on the ribs dimension and spacing, while a deeper rib and a larger number of ribs provide a higher obstruction to concrete to resist the slip of steel bar. It can be seen from Table 2 that the height of rib is 1.17 mm, 1.24 mm and 1.33 mm with a corresponded spacing from 8.54 mm, 10.48 mm and 10.61 mm when the steel bar diameter *d* = 14 mm, 16 mm, and 20 mm. Therefore, the corresponded number of ribs in bond length (5*d*) was 8, 7 and 9. The inconsistent numbers of ribs in the test specimens leads a different bond performance of steel bar with different diameter that is presented on the bond stress-slip curve, which results in the disturbing to the bond-stress distribution along the bond length. However, for the specimens with a fibre volume fraction of 0.8%, 1.2%, 1.6% and 2.0%, an error to the average of the bond strength only was −1.75 to 4.27%, −9.48 to 7.07%, −4.32 to 5.90% and −1.75 to 4.42%. Therefore, the influence of steel bar diameter on the bond strength can be neglected, due to the error of test results was within a limit of 15% specified^9^.

### Effect of SFRC strength

**Figure 9:**
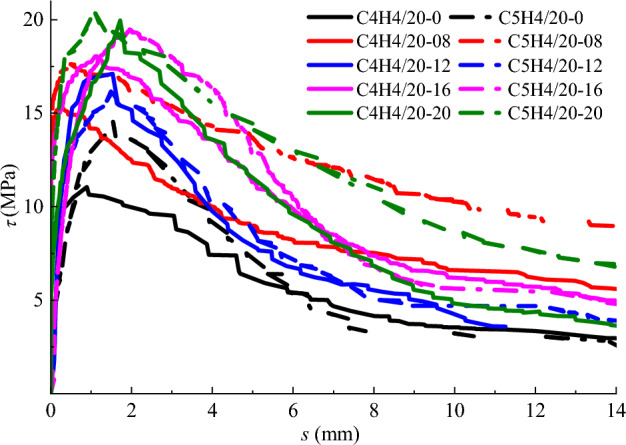
Bond stress-slip curves of specimens with different concrete strength.

The bond stress-slip curves of specimens with different SFRC strength are exhibited in Fig. 9, in which the specimens are divided into a group with the same steel bar and fibre volume fraction. With the increase of concrete strength, the elastic stage of ascending portion of the curves became longer, because of the accordingly increased chemical adhesiveness and the interlocking effect. This is identical to the study of Garcia-Taenga et al.^21^ that a higher concrete compressive strength generates a higher bond strength. Meanwhile, compared with the specimens with C40 SFRC strength grade, the specimens with C50 SFRC strength grade exhibit a steeper descending slope of the bond stress-slip curve. This indicates a fast reduction of the residual bond strength at descending stage of the bond-slip curve, although the absolute values remain at a higher level.

### Effect of fibre volume fraction

**Figure 10:**
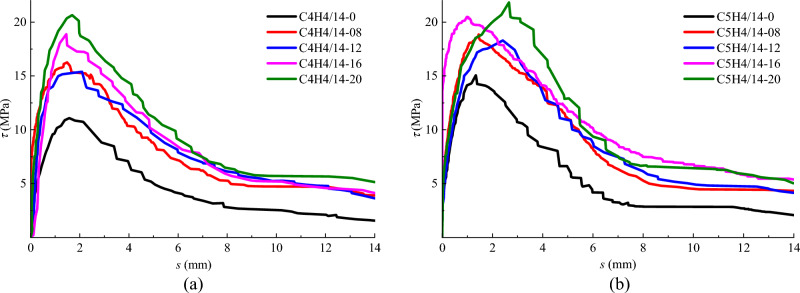
Bond stress-slip curves of specimens with different volume fraction of steel fibre. (**a**) C40 concrete and *d*=14 mm (**b**) C50 concrete and *d*=14 mm.

The bond stress-slip curves of specimens with different fibre volume fraction are shown in Fig. 10, in which the specimens are divided into a group with the same diameter of steel bar in the same strength grade of SFRC. A slight influence of steel fiber on the elastic ascending portion of bond stress-slip curve occurred, due to the bonding stress mainly came from the chemical adhesiveness on the interface of steel bar in SFRC. With the increase of the bond slip, the slope of the ascending portion trends to be smaller until it reached the peak bonding stress. This attributes to the interlocking effect of steel fibres which increases the bond stiffness between ribs of steel bar^24,26^. Once the steel bar presented an obvious slip, the bond-slip curve drops down with a deeper slope, followed by a gradually descending offstage with a slowly decreasing of bond-stress at a larger slip rate. With the increase of fibre volume fraction, the bond strength and peak-slip behave an obvious growth, except for the specimen C5H4/14-16 presents a less slip at the ascending portion of the curve. For the two groups of specimens coded in Fig. 8, the bond strength had an increase of 26.7% and 15.7% for the SFRC with a fibre volume fraction increased from 0.8 to 2.0%, respectively. However, compared with that for referenced conventional concrete, the bond strength creates an increase of 24.4% and 25.2% for the SFRC with a fibre volume fraction of 0.8%. This indicates that the presence of steel fibres inherently improves the bond behavior of steel bar in concrete.

Meanwhile, the residual bond strengths of specimens exhibit an increase with the fibre volume fraction, although a steeper reduction appears at the descending portion of bond stress-slip curve. A larger area under the bond-slip curve, representing a significantly increase of the energy dissipation during the bond-slip, created with the increase of fibre volume fraction. This indicates the contribution of steel fibres to the bond-slip toughness due to the increased confinement of steel fibres around the interface of steel bar in SFRC^26^, and the interlocking between steel fibers and steel bar ribs^30^.

### Prediction of bond strength and peak-slip

The test results of bond-strength (*τ*_u_) and peak-slip (*s*_u_) are shown in Table 4. Based on above discussion of the bond stress-slip behaviours with influencing factors, the variation of *τ*_u_ with the diameter of steel bar is first analyzed. Results show that with the increase of the diameter of steel bar from 14 to 20 mm, *τ*_u_ presented a flat tendency despite of the dispersion of test results. Similarly, the tested specimens were assembled in a group with the same fibre volume fraction. This once again indicated that in condition of *l*_a_ = 5*d,* the changes of diameter and strength of steel bar had a slight effect on *τ*_u_.

**Table 4:** Test results of bond strength and pike-slip.

Trial no.	*s*_u_ (mm)	*τ*_u_ (MPa)	*τ*_u_/*τ*_u0_
C4H4/14-0	1.57	13.08	1.00
C4H4/14-0.8	1.49	16.27	1.24
C4H4/14-1.2	2.09	15.39	1.18
C4H4/14-1.6	1.45	18.88	1.44
C4H4/14-2.0	1.69	20.65	1.58
C4H4/20-0	0.89	13.05	1.00
C4H4/20-0.8	0.24	15.43	1.18
C4H4/20-1.6	1.10	18.04	1.38
C4H4/20-2.0	1.72	19.97	1.53
C5H4/14-0	1.34	15.06	1.00
C5H4/14-0.8	1.45	18.86	1.25
C5H4/14-1.2	2.41	18.30	1.22
C5H4/14-1.6	0.98	20.48	1.36
C5H4/14-2.0	2.66	21.83	1.45
C5H4/16-0	1.25	15.13	1.00
C5H4/16-0.8	1.38	17.77	1.17
C5H4/16-1.2	1.67	19.12	1.26
C5H4/16-1.6	1.87	21.12	1.39
C5H4/16-2.0	1.72	20.54	1.36
C5H4/20-0	1.54	14.54	1.00
C5H4/20-0.8	0.49	17.63	1.21
C5H4/20-1.2	1.49	16.17	1.11
C5H4/20-1.6	1.95	19.48	1.34
C5H4/20-2.0	1.10	20.35	1.40

**Figure 11:**
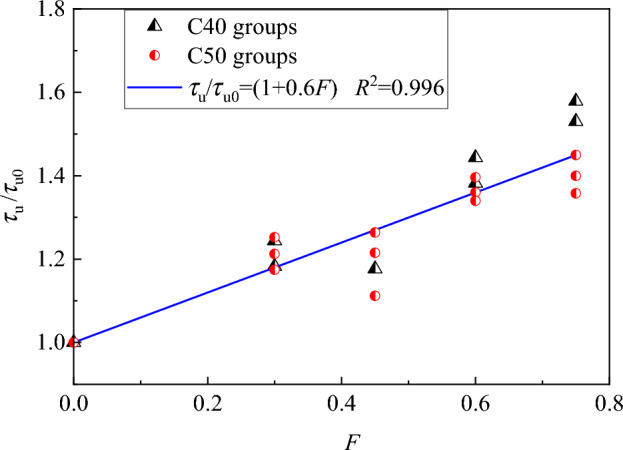
Relationship between *τ*_u_/*τ*_u0_ and *F.*

Meanwhile, the bond strength was higher with a higher strength of SFRC. The bond strength of steel bar in C50 concrete increased 13.3% in average than that of C40 concrete. Disregarding the impact of the diameter of steel bar, the bond strength ratio of steel bar in SFRC with and without steel fibres is depicted in Fig. 11 with a correlation to the fibre factor. It can be seen that despite a large scatter of test data, a similar tendency is presented for the bond strength ratio with the fibre factor, which indicates a less coupling effect between SFRC strength and fibre factor on the bond strength. This is consistent to the study of Hou et al.^25^ that the steel fibre effect is correlated with the fibre factor and fibre distribution uniformity. Based on the test data, the following formula can be gotten by a linear fitness,

4 $$\tau_{{\text{u}}} = \left( {1 + 0.6F} \right)\tau_{{{\text{u0}}}}$$

Comparing Eq. (4) with Eq. (1), the reinforcing of steel fibre on bond strength is weaker than that on tensile strength, as the reinforcing coefficient of 0.60 for bond strength is smaller than that of 0.74 for tensile strength. This attributes the different strengthening effects of steel fibers, the reinforcing contribution to the bond strength relies in certain degree on the increase of tensile strength to provide an effective confinement of steel fibres around the interface of steel bar and the interlocking of SFRC between ribs of steel bar^25,26,42^.

**Figure 12:**
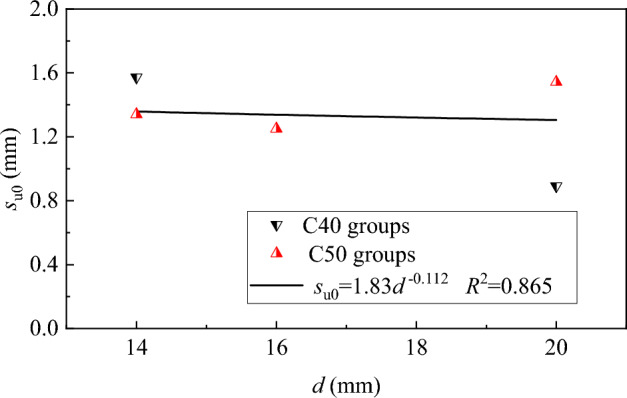
Relationship between *s*_u0_ and *d.*

The variation of peak-slip *s*_u0_ for steel bar to conventional concrete is shown in Fig. 12. A relationship between *s*_u0_ and *d* can be obtained by a linear fitting analysis, the expression can be written as,

5 $$s_{{{\text{u0}}}} { = }{{{1}{\text{.83}}} \mathord{\left/ {\vphantom {{{1}{\text{.83}}} {d^{0.112} }}} \right. \kern-0pt} {d^{0.112} }}$$

**Figure 13:**
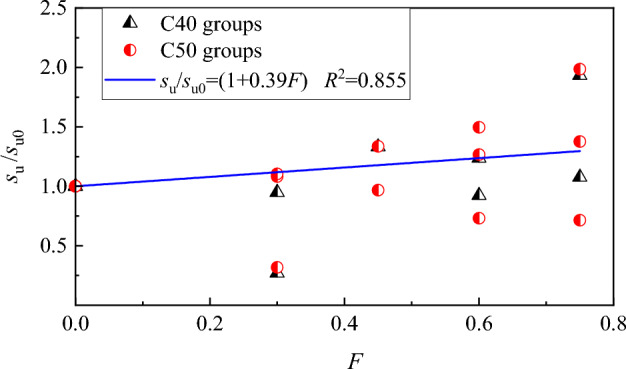
Relationship of *s*_u_ /*s*_u0_ and *F.*

The variation of peak-slip ratio *s*_u_/*s*_u0_ with *F* is shown in Fig. 13, a linear relationship can be obtained by fitting the *s*_u_/*s*_u0_ and *F*, Therefore, combined with Eq. (5), the peak-slip of steel bar in SFRC can be predicted with the equation expressed as,

6 $$s_{{\text{u}}} = \left( {1 + 0.39F} \right)s_{{{\text{u0}}}}$$

### Prediction of anchorage length

Based on the equilibrium of the actions on the bond length of steel bar, with a premise of ensuring that the yield of steel bar is prior to the bond-slip reached the peak-slip, the anchorage length can be calculated by the formula as follows^31^:

7 $$l_{{\text{a}}} { = }\frac{{f_{{\text{y}}} }}{{4\tau_{{\text{u}}} }}d$$

Substitute Eq. (4) into it, the equation transforms as:

8 $$l_{{\text{a}}} { = }\frac{{f_{{\text{y}}} }}{{4\tau_{{{\text{u0}}}} \left( {1 + 0.6\lambda_{{\text{f}}} } \right)}}d$$

If no steel fibre is used for reinforcing conventional concrete, Eq. (8) is degenerated into that used for calculating the anchorage length (*l*_a0_) of steel bar in conventional concrete, that is,

9 $$l_{{{\text{a0}}}} { = }\frac{{f_{{\text{y}}} }}{{4\tau_{{{\text{u0}}}} }}d$$

Therefore, when the strength grade of conventional concrete is determined, the anchorage length (*l*_a0_) of steel bar can be obtained according to relative design codes of concrete structures^23,32,33^. Based on a principle of an equivalent strength grade of SFRC to conventional concrete, the anchorage length of steel bar in SFRC can be obtained using the following equation:

10 $$l_{{\text{a}}} { = }\frac{{l_{{{\text{a0}}}} }}{{\left( {1 + 0.6F} \right)}}$$

According to the specification of Chinese code GB50010^32^, the anchorage length of steel bar in conventional concrete is computed with the Eq. (11a) transferred from Eq. (9),

11a $$l_{{{\text{a0}}}} { = }\alpha \frac{{f_{{\text{y}}} }}{{f_{{{\text{t0}}}} }}d$$

In which the coefficient $\alpha { = }{{f_{{{\text{t0}}}} } \mathord{\left/ {\vphantom {{f_{{{\text{t0}}}} } {4\tau }}} \right. \kern-0pt} {4\tau }}_{{{\text{u0}}}}$, taken as a constant of 0.14 for ribbed steel bar.

With a premise of steel bar in SFRC behaves a similar bond behaviour with the steel bar in conventional concrete, the anchorage length of steel bar in SFRC is computed with Eq. (11b) using the tensile strength of SFRC replacing that of conventional concrete as specified in Chinese code JGJ/T465^28^, that is,

11b $$l_{{\text{a}}} { = }\alpha \frac{{f_{{\text{y}}} }}{{f_{{\text{t}}} }}d$$

Submitted Eq. (11a) into Eq. (11b), the equation can be rewritten as,

12a $$l_{{\text{a}}} { = }\frac{{f_{{\text{t}}} }}{{f_{{{\text{t0}}}} }}l_{{{\text{a0}}}}$$

With the specification of $f_{{\text{t}}} { = }\left( {{1 + 0}{\text{.7}}F} \right)f_{{{\text{t0}}}}$ in Chinese code JGJ/T465^28^, Eq. (12a) can be rewritten as a similar expression of Eq. (10), that is

12b $$l_{{\text{a}}} { = }\frac{{l_{{{\text{a0}}}} }}{{\left( {1 + 0.7F} \right)}}$$

Therefore, when the fibre volume fraction varies from 0.5% to 2.0%, a 1.9–5.3% shorter anchorage length of steel bar in SFRC is given out from Eq. (12b) than that from Eq. (10). This may lead to a lower reliability for the anchorage length of steel bar in SFRC when the tensile strength of SFRC is directly used to replace that of conventional concrete. This difference comes from the lack of test data in developing specification of Chinese code JGJ/T465 to verify the reasonability of Eq. (11b) for the bond of steel bar in SFRC with ingot mill steel fibres. Therefore, it is necessary to revise the specification of anchorage length to meet the actual property.

It can be gotten from this equation that the anchorage length of steel bar in SFRC shortens with the increase of fibre factor, compared to that of steel bar in conventional concrete at an equal strength grade. For instance, using the steel fibre of this study and taken the volume fraction of 0.8–2.0%, the anchorage length of steel bar in SFRC can be shortened by 18–45%. This is convenience for the construction of reinforced concrete structural joints with a shortened anchorage length of steel bar. That is, applied SFRC technology in reinforced concrete structures, the criss-cross issue of many steel bars within the structural joints can be solved, which makes concrete easily cast to rise the concrete quality.

## Conclusions

Considered the influencing factors of SFRC strength grade, fibre volume fraction, and steel bar diameter, an experimental study was carried out on twenty-four groups of specimens using the central pull-out test method. The formulae for predicting the bond strength and peak-slip, and the anchorage length are proposed based on the test results analysis. Conclusions can be drawn as follows:

With a sufficient concrete cover around steel bar and a higher tensile strength of SFRC, all SFRC bonding specimens failed with pull-out of steel bar. The feature of failure was apparently expressed with the steel bar that was pull-in at free end and pull-out at tension end. No yield of steel bar while no splitting of SFRC block took place in this study.

The strength of SFRC was dominant to the bond of ribbed steel bar in SFRC, resulting in the bond strength with an increase of 13.3% when the strength grade of SFRC changed from C40 to C50. The presence of steel fibres generated a positive effect on the bond behavior of steel bar in SFRC. Compared with the referenced conventional concrete specimens, an increase of 19.2% was presented for the bond strength of steel bar in SFRC with a fibre volume fraction of 0.8%, and an increase of 48.0% when the fibre volume fraction is 2.0%. The bond slip is negative to the steel bar diameter, and positive to the fibre volume fraction.

The equations for predicting the bond strength and peak-slip of steel bar in SFRC are proposed, which are associated with the bond strength and peak-slip of steel bar in conventional concrete by considering the effect of fibre factor. And then, the anchorage length of steel bar in SFRC is predicted considered the reinforcing of steel fibre on the bond strength. It is valuable that using the ingot mill steel fibre of this study with a volume fraction of 0.8–2.0%, the anchorage length can be shortened by 18–45%.

Compared with test results of this study, a little shorter anchorage length of steel bar in SFRC is obtained from the specification of Chinese code JGJ/T465. This indicates that a revision is necessary for the reliable anchorage of steel bar in SFRC with ingot mill steel fibres.

## Data Availability

The datasets used and/or analysed during the current study available from the corresponding author on reasonable request.

## List of symbols

Trial no. CsH4/d-*v*_f_: Cs—strength grade, C4 expresses C40, and C5 expresses C50; H4—400 MPa grade hot-rolled ribbed steel bar; d—diameter of steel bar (mm); *v*_f_—volume fraction of steel fibre (%)
*P*: The pull-out force (N)
$\tau$: The average bond stress (MPa)
*τ*_u_: The bond strength of steel bar with SFRC (MPa)
*τ*_u0_: The bond strength of steel bar embedded in concrete with the same matrix of SFRC (MPa)
*f*_t_: The tensile strength of SFRC (MPa)
*f*_t0_: The tensile strength of concrete with the same matrix of SFRC (MPa)
*f*_y_: The yield strength of steel bar (MPa)
*d*: The diameter of steel bar (mm)
*l*_b_: The bonding length of steel bar (mm)
*l*_f_/*d*_f_: The aspect ratio of steel fibre
*v*_f_: The volume fraction of steel fibre
*F*: The fibre factor
*η*_f_: The fibre bonding factor
*s*_u_: The peak-slip for steel bar in SFRC (mm)
*s*_u0_: The peak-slip for steel bar in concrete with the same matrix of SFRC (mm)
*l*_a_: The anchorage length of steel bar in SFRC (mm)
*l*_a0_: The anchorage length of steel bar in concrete with the same matrix of SFRC (mm)
*α*: The coefficient of bond strength

## References

[1] Shakir, Q. M. Response of innovative high strength reinforced concrete encased-composite corbels. *Structures* **25**, 798–809 (2020). 10.1016/j.istruc.2020.03.056

[2] Shang, P. *et al.* Experimental study on external loading performance of large diameter prestressed concrete cylinder pipe. *Buildings* **12**(10), 1740 (2022). 10.3390/buildings12101740

[3] Mostafa Kazemi, M. *et al.* Non-linear behaviour of concrete beams reinforced with GFRP and CFRP bars grouted in sleeves. *Structures* **23**, 87–102 (2020). 10.1016/j.istruc.2019.10.013

[4] Reis, E. D., de Azevedo, R. C., Christoforo, A. L., Poggiali, F. S. J. & Bezerra, A. C. S. Bonding of steel bars in concrete: A systematic review of the literature. *Structures* **49**, 508–519 (2023). 10.1016/j.istruc.2023.01.141

[5] Kim, S.-W. & Yun, H.-D. Evaluation of the bond behavior of steel reinforcing bars in recycled fine aggregate concrete. *Cem. Concr. Compos.* **46**, 8–18 (2014). 10.1016/j.cemconcomp.2013.10.013

[6] Qi, A., Liu, X., Xu, R. & Huang, Y. Bond behavior of steel reinforcement in concrete containing ferronickel slag and blast furnace slag powder. *Constr. Build. Mater.* **262**, 120884 (2020). 10.1016/j.conbuildmat.2020.120884

[7] Leibovich, O. & Yankelevsky, D. Z. Bond behavior in pull-out of a ribbed rebar from concrete with recycled concrete aggregates. *Case Stud. Constr. Mater.* **17**, e01607 (2022).

[8] Shen, D., Shi, X., Zhang, H., Duan, X. & Jiang, G. Experimental study of early-age bond behavior between high strength concrete and steel bars using a pull-out test. *Constr. Build. Mater.* **113**, 653–663 (2016). 10.1016/j.conbuildmat.2016.03.094

[9] GB/T50081-2019. *Standard for Test Methods of Physical and Mechanical Properties on Concrete* (China Building Industry Press, 2019).

[10] Zhao, S., Ding, X., Li, C. & Li, C. Experimental study on bond properties of deformed steel bar to manufactured sand concrete. *J. Build. Mater.* **16**(2), 193–199 (2013).

[11] Gaurav, G. & Singh, B. Analytical investigation in bond of deformed steel bars in recycled aggregate concrete. *J. Sustain. Cem. Based Mater.* **9**, 191–217 (2020).

[12] Liu, G., Dou, X., Qu, F., Shang, P. & Zhao, S. Bond behavior of steel bars in concrete confined with stirrups under freeze–thaw cycles. *Materials* **15**(20), 7152 (2022). 36295219 10.3390/ma15207152 PMC9607287

[13] Li, X., Pei, S., Fan, K., Geng, H. & Li, F. Bending performance of SFRC beams based on composite-recycled aggregate and matched with 500 MPa rebars. *Materials* **13**(4), 930 (2020). 32093065 10.3390/ma13040930 PMC7078638

[14] Zhao, M., Li, J., Xie, Y. M. & Shen, J. Semi-empirical synergetic analysis of the shear capacity of steel fiber reinforced concrete slender beams of rectangular-sections without stirrups. *Eng. Struct.* **285**, 116035 (2023). 10.1016/j.engstruct.2023.116035

[15] Zhao, M., Li, C., Su, J., Shang, P. & Zhao, S. Experimental study and theoretical prediction of flexural behaviors of reinforced SFRELC beams. *Constr. Build. Mater.* **208**, 454–463 (2019). 10.1016/j.conbuildmat.2019.03.037

[16] Shakir, Q. M. & Hanoon, H. K. New models for reinforced concrete precast hybrid deep beams under static loads with curved hybridization. *Structures* **54**, 1007–1025 (2023). 10.1016/j.istruc.2023.05.084

[17] Shakir, Q. M., Hannon, H. K. & Farsangi, E. N. Enhancing the performance of precast hybrid concrete deep beams using curved and arched designs: Experimental investigations. *Structures* **58**, 105371 (2023). 10.1016/j.istruc.2023.105371

[18] Han, R., Zhao, S. & Qu, F. Experimental study on the tensile performance of steel fiber reinforced concrete. *China Civ. Eng. J.* **39**(11), 63–67 (2006).

[19] Ding, X., Li, C., Han, B., Lu, Y. & Zhao, S. Effects of different deformed steel-fibers on preparation and properties of self-compacting SFRC. *Constr. Build. Mater.* **168**, 471–481 (2018). 10.1016/j.conbuildmat.2018.02.162

[20] Yazıcı, S. & Arel, H. S. The effect of steel fiber on the bond between concrete and deformed steel bar in SFRCs. *Constr. Build. Mater.* **40**, 299–305 (2013). 10.1016/j.conbuildmat.2012.09.098

[21] Garcia-Taengua, E., Martí-Vargas, J. R. & Serna, P. Bond of reinforcing bars to steel fiber reinforced concrete. *Constr. Build. Mater.* **105**, 275–284 (2016). 10.1016/j.conbuildmat.2015.12.044

[22] Chu, S. H. & Kwan, A. K. H. A new bond model for reinforcing bars in steel fibre reinforced concrete. *Cem. Concr. Compos.* **104**, 103405 (2019). 10.1016/j.cemconcomp.2019.103405

[23] *fib* MC2010. *Fib Model Code for Concrete Structures*. (Ernst & Sohn Publishing House, 2013).

[24] Li, L. *et al.* Synergistic effects of steel fibres and expansive agent on steel bar-concrete bond. *Cem. Concr. Compos.* **104**, 103380 (2019). 10.1016/j.cemconcomp.2019.103380

[25] Hou, L., Sun, H., Liu, G., Huang, T. & Chen, D. Bond strength of deformed reinforcement embedded in steel fiber reinforced concrete: Influencing factors and prediction model. *Constr. Build. Mater.* **407**, 133436 (2023). 10.1016/j.conbuildmat.2023.133436

[26] de Alencar Monteiro, V. M., Cardoso, D. C. T., de Andrade Silva, F. & Mobasher, B. The influence of steel fibers on the bond slip behavior between rebars and concrete: Experimental and analytical investigation. *Constr. Build. Mater.* **411**, 134357 (2024). 10.1016/j.conbuildmat.2023.134357

[27] Ding, X. *et al.* Prediction of the complete flexural load-deflection curve of self-compacting concrete reinforced with hook-end steel fiber. *Constr. Build. Mater.* **409**, 134003 (2023). 10.1016/j.conbuildmat.2023.134003

[28] JGJ/T465-2019. *Design Code for Steel Fiber Reinforced Concrete Structures* (China Building Industry Press, 2019).

[29] Zhao, M., Li, J. & Law, D. Effects of flowability on SFRC fibre distribution and properties. *Mag. Concr. Res.* **69**(20), 1043–1054 (2017). 10.1680/jmacr.16.00080

[30] Zhao, M., Li, J. & Xie, Y. Effect of vibration time on steel fibre distribution and flexural properties of steel fibre reinforced concrete with different flowability. *Case Stud. Constr. Mater.* **16**, e01114 (2022).

[31] Zhang, X., He, F., Chen, J., Yang, C. & Xu, F. Orientation of steel fibers in concrete attracted by magnetized rebar and its effects on bond behavior. *Cem. Concr. Compos.* **138**, 104977 (2023). 10.1016/j.cemconcomp.2023.104977

[32] GB500102010. *Code for Design of Concrete Structures* (China Building Industry Press, 2010).

[33] ACI 318-19. *Building Code Requirements for Structural Concrete*. (ACI Committee 318, ACI, 2019).

[34] Zhao, M. *Study on Shear Behaviours of Reinforced High-Performance SFRC Beams* (RMIT University, 2023).

[35] GB 175-2007. *Common Portland Cement* (China Standard Press, 2007).

[36] GB/T 1596-2017. *Fly Ash for Cement and Concrete* (China Standard Press, 2017).

[37] JGJ 52-2006. *Standard for Technical Requirements and Test Method of Sand and Crushed Stone (or Gravel) for Ordinary Concrete* (China Building Industry Press, 2006).

[38] JG/T472-2015. *Steel Fiber Reinforced Concrete* (China Standard Press, 2015).

[39] JGJ55-2011. *Specification for Mix Proportion Design of Ordinary Concrete* (China Building Industry Press, 2011)

[40] Ding, X., Zhao, M., Li, J., Shang, P. & Li, C. Mix proportion design of self-compacting SFRC with manufactured sand based on the steel fiber-aggregates skeleton packing test. *Materials* **13**(12), 2833 (2020). 32599835 10.3390/ma13122833 PMC7344632

[41] GB50080-2016. *Standard for Test Method of Performance on Ordinary Fresh* (China Building Industry Press, 2016).

[42] Zhao, M. *et al.* Bond of ribbed steel bar in steel fiber reinforced expanded-shale lightweight concrete. *Buildings* **11**(12), 582 (2021). 10.3390/buildings11120582

